# Patterns and trajectories of inequality in physical activity from childhood to adolescence in Kazakhstan

**DOI:** 10.1016/j.pmedr.2024.102729

**Published:** 2024-04-16

**Authors:** Kwok Ng, Assel Adayeva, Shynar Abdrakhmanova, Stephen Whiting, Julianne Williams, Tatyana Slazhnyova, Galina Kaussova

**Affiliations:** a Physical Activity for Health Research Centre, Department of Physical Education and Sport Sciences, University of Limerick, Ireland; b Faculty of Education, University of Turku, Finland; c Institute of Innovation in Sports Science, Lithuanian Sports University, Lithuania; d Faculty of Sport and Health Sciences, University of Jyväskylä, Finland; e National Center of Public Health of the Ministry of Health, Kazakhstan; f Kazakhstan Medical University "High School of Public Health", Kazakhstan; g Special Initiative on NCDs and Innovation, WHO Regional Office for Europe, Copenhagen, Denmark

**Keywords:** Family structure, Urban, Rural, Teenager, Children

## Abstract

**Objective:**

Sociodemographic differences in physical activity (PA) levels during the transition from childhood to adolescence, particularly in Central Asian countries, is lacking. In this study, we examine individual, family and environmental sociodemographic variables associated with PA among children and young adolescents in Kazakhstan.

**Study design:**

Secondary data analyses of two nationally representative cross-sectional studies administered by parents of children (aged 7–9 y) and by self-report surveys (adolescents aged, 11–15 y) .

**Methods:**

The 2020 Childhood Obesity Surveillance Initiative (COSI) and 2018 Health Behaviour in School-aged Children (HSBC) studies were used. Predictors of daily PA by individual (gender, grade, weight status), family (family composition, family employment, family wealth), and environment (school location) factors were analysed through binary logistic regressions after adjusting for screen time.

**Results:**

Among both children and adolescents, males were more physically active than females. Daily PA among children was positively associated with high family employment (OR = 1.23, CI = 1.03–1.48) or living in an urban location (OR = 0.58, CI = 0.49–0.70). Daily PA was associated with low family wealth, two-parent families (OR = 1.25 CI = 1.08–1.44) or living in a rural location (OR = 1.18 CI = 1.04–1.34) among adolescents.

**Conclusion:**

In Kazakhstan, the trajectory of children’s and adolescent’s PA levels, differed according to individual, family and environmental characteristics , suggesting a need for age-specific, targeted interventions to promote PA, appropriate policies that promote programs in schools, communities, and development of infrastructure for physically active lifestyles.

## 1 Introduction

Regular physical activity in childhood and adolescence is important for healthy growth and development as well as for the prevention of non-communicable diseases. The physical, social and mental health benefits of PA are well documented (Janssen and LeBlanc, 2010). The uptake of physical activity during adolescence is a predictor of regular physical activity during adulthood (Hayes et al., 2019, Telama et al., 2014). Yet, PA levels decline during the transition from childhood to adolescence (Pate et al., 2022). National estimates of participating in daily PA from Kazakhstan range from 69 % amongst 9y olds to 31 % amongst 15 year olds (Ng et al., 2019). Declining PA levels at such early ages of a person’s life can lead to early onset of disability and non-communicable diseases (Murray et al., 2020), hence to plan, implement and evaluate programs, it is essential to understand other sociodemographic factors associated with PA levels (Hämäläinen et al., 2016).

Kazakhstan is an upper-middle income country with a relatively young population (The World Bank, 2022). In general, Kazakhstan is dynamically developing in socio-economic terms, undergoing through a number of challenges: political changes, economic diversification, while undertaking efforts to enhance the economic well-being and inequality, and improving access to education and healthcare (Kazakhstan-2050 Strategy, 2012). Structural inequities, such as individual characteristics, wealth, physical environment, and housing exist in health and are ever present concerning physical activity participation (Baciu et al., 2017).

At the individual level, more males are reported to take part in daily PA than females (Guthold et al., 2020). Where a person lives defines the challenges and opportunities towards their access to PA facilities, transport, air quality, and costs associated with participation, thus influencing behavioural patterns (Rutter et al., 2019). The associations between neighbourhoods and PA levels are context-specific. For example, in a study on adolescents on a Pacific island, rural adolescents were more active than urban peers (Wattelez et al., 2021), whereas, through a review of the literature on children in China, Lu and colleagues (Lu et al., 2017) concluded that rural children were less physically active compared to urban dwellers.

In various studies, associations have been reported between higher socioeconomic family status and increased levels of PA (Falese et al., 2021). Furthermore, family structure appears to be an important link with children’s PA levels, where two parents in a family was associated with increases in overall PA, organized sport participation, and reduced screen time levels of adolescents (Langøy et al., 2019), whereas children in single-parent families are more likely to experience challenges to participate in organized physical activities (Singhammer et al., 2015). Such findings based on sociodemographic differences have, so far, not been explored in the context of Kazakhstan.

The decline in PA levels from childhood to adolescence is a major concern for public health officials. Available data from WHO European Childhood Obesity Surveillance Initiative (COSI) cross-sectional study (2018–2020) indicated that 87.0 % of 6–9 year old primary school children in 27 countries, including Kazakhstan, were physically active on a daily basis (World Health Organization, 2022). Levels drop substantially when it comes to adolescents. According to 2018 Health Behaviour in School-aged Children (HBSC) study, only 19.0 % of 11–15 year old adolescents met the recommended daily 60 min of moderate-to vigorous PA (MVPA) level (Inchley et al., 2020).

Inequalities also exist among adolescents, although there is a knowledge gap in how the sociodemographic factors develop during the transition from child to adolescence. Knowing the sociodemographic inequalities in PA during childhood through adolescence could identify required public health strategies to address and maintain equality of PA opportunities at this crucial habit-forming life stage. Yet, there are no known large-scale studies that have comprehensively covered the trajectory of these inequalities from childhood to adolescents, therefore, studies need to be combined. There are, however, some challenges when combining studies as instruments and methodology could differ, making comparisons of limited utility. Yet, the benefits of gaining insights from combining studies outweigh the limitations. Hebestreit and colleagues (Hebestreit et al., 2022) suggested the need for adolescent lifestyle behaviour items to be harmonized to increase the comparability of the existing surveillance systems.

Therefore, the aim of the paper is to explore the sociodemographic factors of daily PA among children and adolescents. Two overarching research questions are examined in this paper; 1. What are the sociodemographic inequalities towards daily PA, according to gender, grade, urbanicity, parental employment, family material wealth, family structure, BMI and screen time among children and adolescents in Kazakhstan? and 2. How do these sociodemographic inequalities differ across childhood and adolescence?

## 2 Methods

### 2.1 Study design

This study combines two international studies that were designed to produce nationally representative data. The WHO European Childhood Obesity Surveillance Initiative (COSI) study is a cross-sectional, of primary schoolchildren and the Health Behaviour in School-aged Children (HBSC) is a WHO collaborative cross-national study of adolescent health and well-being of school-age children in early adolescence. Boarding schools and specialized schools for children with special needs constituted less than 1.0 % of the target population and were excluded from the sampling frame. A two-stage cluster sampling design was used in both studies. Schools are the primary sampling units and classes as the secondary sampling units. The samples for both studies in Kazakhstan are stratified by urban and rural environments. Data were collected by the National Center of Public Health and the standard methodology of the international protocols (World Health Organization, 2022, Inchley et al., 2020).

### 2.2 Setting

Data collection took place in all 17 administrative regions of the country, namely 14 oblasts and three major cities in September-December in 2017 for HBSC and 2020 for COSI. In the COSI study, a paper-based questionnaire was distributed via children to their parents to fill out and return to a teacher. In the HBSC study, students completed a self-report survey during a class administered by a teacher. In the present paper, we refer to children as primary school children from the COSI study, and we refer to adolescents from the HBSC study.

### 2.3 Participants

The COSI study included students in 2nd and 3rd grade (7–8 y and 8–9 y olds respectively), and the HBSC study incorporated students in 5th, 7th and 9th grades (11y, 13y, and 15y olds, respectively). In the COSI study, there were 153 general education schools (urban n = 81, rural n = 72) and in the HBSC study, there were 110 general education schools (urban n = 54, rural n = 56) in the final sample. All present children from the chosen classes were eligible for participation. Exceptional children outside the age groups, absentees, or those without returned parental forms were excluded from the analyses.

Children who studied in the first grade (n = 422) were excluded from the data set of the COSI study, because the coverage of this grade category does not correspond to the scope of our study. In addition, children aged 8 years in the HBSC study (n = 11) were excluded from the sample of children in the HSBC study as outliers from the HBSC age groups of 11y, 13y and 15y olds.

### 2.4 Independent variables

In the COSI study, registries were used for coding data for gender, school grade, and school location. Furthermore, the children’s height and weight were measured according to a standard procedure to the nearest 0.1 cm and 0.1 kg, respectively, by trained fieldworkers. Children were in light clothing and weight was adjusted by subtracting the weight of clothes. Parents answered questions about their employment circumstances, family structure, as well as the child’s behaviours.

The HBSC study was completed solely through self-report questions. Although some recall biases have been reported, the use of BMI categories has been demonstrated to be suitable when reported on national representative samples (Inchley et al., 2020). After calculating the BMI through weight (kg) divided by height squared (m2), values below −5 or above + 5 z-scores relative to the 2007 WHO growth reference (De Onis, 2007) median were recoded as missing. A summary of the variables and the harmonization are outlined in Table 1.

**Table 1:** Summary of the factors that measured inequalities across Kazakhstan COSI 2020 and HBSC 2018 studies and the harmonisation recoding.

Factor		COSI 2020	Response Recoding	HBSC 2018	Response Recoding
*Individual factors*					
	Gender	Registry data	1 = male, 2 = female	Self-report	1 = male, 2 = female
	Grade	School registry data	8th = 1, 9th = 2	self-report	5th = 3, 7th = 4, 9th = 5
	Age	date of birth from school registry, if missed, from parental form. Age was calculated by subtracting date of birth from date of child measurement, in months, than converted to years		Age was	
	Body mass	calculated as kg/m2 and use of WHO cut-offs.	1 = underweight, 2 = normal weight, 3 = overweight, 4 = obese	calculated as kg/m2 and use of WHO cut-offs.	1 = underweight, 2 = normal weight, 3 = overweight, 4 = obese

*Individual behaviour*					
	Total screen time	Time spent watching TV or playing on computer, tablet, smartphone and other devices on weekdays and weekends (except for device based fitness games). (5*week)+(2*weekend)/7 = weighted average	1 = up to 3 h/day 0 = 3 h/day or more	Time spent on separate devices on week days and weekends; 1. TV, 2. Computer, 3. Playing computer games. (5*week)+(2*weekend)/7 = weighted average	1 = up to 3 h/day 0 = 3 h/day or more

*Family factors*					
	Family Wealth	1 = trouble making ends meet, 2 = barely making ends meet, 3 = pass the month without serious problems, 4 = we easily pass the month with our earnings.	1 = low family wealth 2 = medium family wealth 3 = high family wealth	Family affluence scale III, 6 items on material wealth in the family home (Hartley et al., 2016 Mar). Sum score of the 6 items and converted into a rank score.	1 = lowest 33 % (low wealth) 2 = middle 33 % (medium wealth) 3 = highest 33 % (high wealth)
	Family Composition	Father, mother, stepfather or stepmother living in the primary home	1 = two parent family 2 = single parent family	Father, mother, stepfather or stepmother living in the primary home	1 = two parent family 2 = single parent family
	Parental employment	Parental employment status during the last six months. Unemployed is where only one parent is unemployed. When both parents within intact families are working, or single-parent is working	1 = unemployed 2 = employed	Unemployed is where only one parent is unemployed. When both parents within intact families are working, or single-parent is working.	1 = unemployed 2 = employed

*Environment*					
	School location	Registries from sample	1 = urban, 2 = rural	Registries from sample	1 = urban, 2 = rural

COSI = WHO European Childhood Obesity Surveillance Initiative; HBSC = Health Behaviour in School-age Children.

### 2.5 Dependent variable

PA within COSI study was based on parental responses on their child’s time spent playing vigorously. The answer options were categorised into two groups: (1) not daily PA, playing vigorously for less than one hour a day, (2) daily PA, playing vigorously for one hour or more hours a day.

PA in the HBSC study was measured for a single-item self-report item that included a brief introduction to the definitions of PA, with examples, followed by a question of how many days in the past 7 days they were physically active for at least 60 min per day. The item has acceptable test–retest reliability (Ng et al., 2019) and validity against an accelerometer (Hardie-Murphy et al., 2015). Response options were recoded into two groups: (1) not every day (those who indicated 0–6 days per week) and (2) daily (those who indicated 7 days per week).

### 2.6 Study size

Following data collection and cleaning data from missing cases, the data were weighted against the statistical data on the number of children studying in general education schools in Kazakhstan from the National Education Database for 2018 by grade (2, 3, 5, 7, 9 grades), gender (male/female) and place of residence (urban/rural). All analyses were carried out with the weights applied. All missing data was tested for sample biases by testing differences between independent variables and PA levels. No statistically differences were detected, hence the missing cases were removed from the analysed data set. No values were imputed.

### 2.7 Statistical methods

For the descriptive statistics, frequency analysis was performed. To further explore how the independent variables differed within COSI and HBSC studies, we used the Chi-square test of independence. To analyse the influence of one or more factors on the outcome (daily PA), we applied binary logistic regression separately for the COSI and HBSC study. In the single-factor analysis, we estimated the effect of each factor separately by calculating the odds ratio (OR) and 95 % confidence intervals (CI) for each factor. In the multifactor analysis, we accounted for the simultaneous effect of multiple factors on the outcome by calculating the OR and 95 % CI and reported on the multifactor analyses in the results.

## 3 Main results

The final cleaned unweighted dataset included children (n = 6429) from the COSI study of 7–9-year-olds (2nd grade n = 3244; 3rd grade n = 3185) and adolescents (n = 6537) from the HBSC study of 11y (5th grade n = 2192), 13y (7th grade, n = 2125), and 15y olds (9th grade n = 2220). The sample is stratified by urban and rural strata.

### 3.1 Descriptive summary

After applying sample weights to the data, the cleaned weighted data consisted of results from 12,965 school-age children and adolescents from 2nd (n = 3047), 3rd (n = 3021), 5th (n = 2528), 7th (n = 2334) and 9th (n = 2035) grades. Details of the independent variables by gender is presented in Table 2.

**Table 2:** Characteristics of children and adolescents (weighted) and differences by gender from Kazakhstan COSI 2020 and HBSC 2018 studies.

	COSI 2020			X2	HBSC 2018			X2	Total
Variables	Total (n = 6068)	Males (n = 3156)	Females (n = 2912)	p	Total (n = 6897)	Males (n = 3508)	Females (n = 3389)	p	Total (n = 12965)
Grades				0.199				0.177	
2nd	50.2	49.4	51.1		−	−	−		
3rd	49.8	50.6	48.9		−	−	−		
5th	−	−	−		36.7	36.3	37.1		
7th	−	−	−		33.8	34.9	32.8		
9th	−	−	−		29.5	28.9	30.2		
Body weight categories (%)	n = 6057	n = 3146	n = 2911	<0.001	n = 6154	n = 3147	n = 3007	<0.001	n = 12211
Thin	5.7	5.8	5.6		10.5	9.3	11.7		8.1
Normal	73.9	70.9	77.1		77.3	75.4	79.4		75.6
Overweight	12.2	12.9	11.4		8.8	10.7	6.9		10.5
Obese	8.3	10.4	6.0		3.3	4.6	2.0		5.8
Location	n = 6068	n = 3156	n = 2912	0.932	n = 6897	n = 3508	n = 3389	0.045	n = 12965
Urban	55.2	55.3	55.2		49.9	48.7	51.2		52.4
Rural	44.8	44.7	44.8		50.1	51.3	48.8		47.6
Family composition (%)	n = 5183	n = 2671	n = 2512	0.959	n = 6569	n = 3331	n = 3238	0.292	n = 11752
Two parent family	87.9	87.9	87.9		73.5	74.1	72.9		79.9
Single parent family	12.1	12.1	12.1		26.5	25.9	27.1		20.1
Family material wealth n (%)	n = 5334	n = 2744	n = 2590	0.737	n = 6744	n = 3427	n = 3317	<0.001	n = 12078
Low	32.1	32.5	31.6		28.6	26.4	30.8		30.1
Medium	35.7	35.3	36.1		33.7	33.6	33.7		34.6
High	32.2	32.1	32.2		37.8	40.0	35.5		35.3
Parental employment (%)	n = 4886	n = 2501	n = 2385	0.460	n = 6374	n = 3226	n = 3148	0.057	n = 11260
Unemployed*	51.5	51.0	52.1		31.7	30.6	32.8		40.3
Employed	48.5	49.0	47.9		68.3	69.4	67.2		59.7
Combined screen time (%)	n = 5137	n = 2645	n = 2492	0.171	n = 5562	n = 2716	n = 2846	0.002	n = 10699
3 h/day and more	10.8	11.4	10.2		60.2	62.2	58.2		36.5
up to 3 h/day	89.2	88.6	89.8		39.8	37.8	41.8		63.5

COSI = WHO European Childhood Obesity Surveillance Initiative; HBSC = Health Behaviour in School-age Children *Unemployed when at least one parent is unemployed in the house.

### 3.2 Prevalence of daily 60 min physical activity

The majority of 2nd and 3rd grade children in Kazakhstan were physically active, daily for at least 1 h/day. Gender differences were statistically significant for 3rd grade (p = .013), where more males were active than females (Table 3). Levels of daily PA were halved for the adolescents, although gender differences (males more active than females) were only statistically significant for 9th graders (p < .001). The gender inequality in daily PA increased 2.3 times with grade, (notably, 3rd grade difference = 3.3 %, 9th grade difference = 7.7 %).

**Table 3:** Prevalence of daily PA at least 60 min per day among children and adolescents by gender and grade from Kazakhstan COSI 2020 and HBSC 2018 studies.

Grades	Total		Males		Females		p (Chi-Square)
Daily MVPA (60 min + )	n	%	n	%	n	%	
*COSI 2020*							
2nd grade (7 year old)	2342	87.9	1190	88.3	1151	87.5	0.52
3rd grade (8 year old)	2275	85.9	1222	87.5	1053	84.2	0.013

*HBSC 2018*							
5th grade (11 year old)	848	35.1	432	35.5	416	34.6	0.66
7th grade(13 year old)	816	36.3	429	36.7	387	35.9	0.68
9th grade(15 year old)	626	31.6	350	35.5	276	27.8	<0.001
Total	6907	57.8	3623	59.3	3283	56.2	0.001

COSI = WHO European Childhood Obesity Surveillance Initiative; HBSC = Health Behaviour in School-age Children.

### 3.3 The sociodemographic inequalities of MVPA stratified by children and adolescents

Table 4 presents the results of binary logistic regressions exploring the associations of daily PA with the independent variables stratified for children (COSI study) and for adolescents (HBSC study). For adolescents, limiting screen time up to 3 h/day (OR = 1.20, 95 %CI = 1.06–1.36) (vs screen time more than 3 h/day) was associated with daily MVPA, but this was not statistically significant among children in the COSI study.

**Table 4:** Odds ratios and 95 % confidence intervals of sociodemographic factors associated with physical activity among children (COSI 2020) and adolescents (HBSC 2018) in Kazakhstan.

		COSI 2020		HBSC 2018	
		OR (95 % CI)	p	OR (95 % CI)	p
Gender					
	Females	1.0 (reference)		1.0 (reference)	
	Males	**1.25 (1.05**–**1.49)**	**0.012**	**1.14 (1.01**–**1.29)**	**0.033**

School grade					
	2nd grade (7y)	1.0 (reference)		−	
	3rd grade (8y)	0.85 (0.71–1.01)	0.068	−	
	5th grade (11y)	−		1.0 (reference)	
	7th grade (13y)	−		1.03 (0.89–1.20)	0.67
	9th grade (15y)	−		0.88 (0.75–1.02)	0.095

Urbanization					
	Rural	**0.58 (0.49**–**0.70)**	**<0.001**	**1.18 (1.04**–**1.34)**	**0.012**
	Urban	1.0 (reference)		1.0 (reference)	

Body weight categories					
	Thin	1.35 (0.85–2.16)	0.21	1.11 (0.73–1.66)	0.63
	Normal	1.29 (0.94–1.76)	0.12	1.32 (0.92–1.90)	0.14
	Overweight	1.33 (0.90–1.98)	0.16	1.20 (0.80–1.82)	0.38
	Obese	1.0 (reference)		1.0 (reference)	

Family Material Wealth					
	Low	1.0 (reference)		1.0 (reference)	
	Medium	0.85 (0.68–1.06)	0.15	**0.85 (0.73**–**0.99)**	**0.033**
	High	0.82 (0.66–1.02)	0.076	**0.72 (0.62**–**0.84)**	**<0.001**

Family composition					
	Single parent	1.0 (reference)		1.0 (reference)	
	Two parents	0.98 (0.73–1.31)	0.9	**1.25 (1.08**–**1.44)**	**0.033**

Parental employment					
	Unemployed	1.0 (reference)		1.0 (reference)	
	Employed	**1.23 (1.03**–**1.48)**	**0.024**	1.02 (0.90–1.17)	0.75

Screentime					
	3 h/day +	1.0 (reference)		1.0 (reference)	
	>3h/day	0.74 (0.53–1.02)	0.065	**1.20 (1.06**–**1.36)**	**0.005**

COSI = WHO European Childhood Obesity Surveillance Initiative; HBSC = Health Behaviour in School-age Children; OR = Odds ratios; CI = 95 % Confidence Intervals.

#### 3.3.1 Individual level factors

Primary school males (OR = 1.25, 95 %CI = 1.05–1.49) and adolescent males (OR = 1.14, 95 %CI = 1.01–1.29) were more likely to report PA 60 min or more in a day than females. There were no statistical differences in PA between 2nd and 3rd grade children, or between 5th, 7th and 9th graders. That is to say, there was no trajectory to report from grade within school levels.

#### 3.3.2 Family factors

There were various ways family factors were associated with daily PA for children and adolescents, Adolescents living in medium (OR = 0.85, 95 %CI = 0.73–0.99) and high (OR = 0.72, 95 %CI = 0.62–0.84) affluent families reported doing less daily PA than adolescents living in a low affluence family. In terms of parental employment, children from families with high parental employment (OR = 1.23, 95 %CI = 1.03–1.48) were more likely to report daily PA than children with low parental employment levels. Adolescents from intact (two parent) families (OR = 1.25, 95 %CI = 1.08–1.44) (vs being from single parent family) were associated with daily PA.

#### 3.3.3 Environmental factors

Children residing in rural settings (OR = 0.58, 95 %CI = 0.49–0.70) were less likely to participate in daily PA than children from urban areas. This was the opposite for adolescents, where the odds ratios for daily PA for residing in rural areas were positive (OR = 1.18, 95 %CI = 1.04–1.34) compared to peers living urban areas.

## 4 Discussion

In Kazakhstan, individual, family and environmental inequalities to physical activity were not consistent between childhood and adolescence. Males were more physically active in both age groups. Interestingly, children of schools in urban environments were more physically active compared to those in rural environments, whereas among adolescents the findings were opposite. Other sociodemographic factors, such as school grade, family composition, family wealth, and parent employment were inconsistent between the two groups.

High proportions of primary school children took part in daily physical activity, yet when it came to young adolescents, the levels dropped to approximately a third of adolescents. Amongst children, daily PA increased from 71 % during the 2015–2017 COSI data collection round (Whiting et al., 2021). The substantial drop from children to adolescence may be related to measure issues, because COSI data was based on parent reports, and HBSC was based on self-report, generating different recall and comprehension biases between surveys (Rebholz et al., 2014). Despite these differences, the decline of PA levels between pupils in primary and secondary schools (Mikalsen et al., 2020) has been well reported. Unexpectedly, there was stability of PA levels between age groups within the COSI and the HBSC studies. Global data suggests that PA levels decline from 11y to 15y olds (Guthold et al., 2020). The lack of decline from Kazakhstan does not seem to be an anomaly, as similar results were reported from the 2022 HBSC study (Rakic et al., 2021) which made Kazakhstani adolescents ranked the highest among 13- and 15-year olds (Inchley et al., 2020).^1^ The high rates of daily PA among Kazakhstani could be related to the mandate of three physical education lessons per week for all year groups (Nurlanov et al., 2017), but more reasons need to be explored.

The only consistent sociodemographic inequality in PA across the transition from childhood to adolescence was gender. Males are more physically active than females and is observed worldwide (Guthold et al., 2020). Yet, when examining the sample by year group, the difference was not always statistically significant. Examples of lack of gender differences have been reported after removing the comparison between daily MVPA from the sample (Ng et al., 2019), suggesting the need for programs where “any move counts” (WHO, 2018) and considerations for gender specific activities.

In our study, intact families were associated with daily MVPA among adolescents but not for children. This could be related to the common multigenerational family structures in Kazakhstan households. Approximately 60 % of families depend on the grandparents to participate in the upbringing of children, even in single-parent families (Parker et al., 2022), thus negating possible inequalities for children. In other studies, no association between parental education, employment status and perceived family wealth with daily active play were found in Kazakhstan families (Musić Milanović et al., 2021). Yet, for adolescents, two-parent families have more opportunities for PA due to the possible support required for structured activities (Langøy et al., 2019, Whiting et al., 2021).

Higher family wealth may be a risk factor in Kazakhstan, which contrasts reports from previous international studies (Chzhen et al., 2018, Pearson et al., 2022). Adolescents from higher family affluence were three times more likely to report daily PA than lower family affluence families (Ke et al., 2022), with the influence of wealth on availability to pay for participation in extra activities (Sigmundová et al., 2019). Yet, family wealth is typically associated with more technology at the house and a higher likelihood of travel by private motorised vehicle (Currie et al., 2024). This in turn contributes to more opportunities for sedentary behaviours, and thus, according to the displacement theory, reduces time spent being physically active (Mutz et al., 1993). A careful balance of being physically active and use of technology is needed in the Kazakhstan region as human development gets more advanced over the years.

Between children and adolescents, there was an opposite association for the environmental factor, as measured by schools in urban or rural areas. Similar to data on Chinese children, thosein urban environments were more physically active than those in rural environments, but results were inconclusive for adolescents (Lu et al., 2017). The lower levels are expected given with a lack of sports infrastructure available in rural areas, and children’s health behaviours being more influenced by their family than peers (Patton et al., 2016). Whereas, the local communities in rural areas have been reported as key determinants for PA among Kazakhstani adolescents (Brown Hajdukova et al., 2017). Similar results were reported in Brazil, where rural adolescents, participate more in agricultural activities and had higher levels of PA compared to their urban peers (Regis et al., 2016).

## 5 Limitations

This study has some limitations when interpreting the results. Harmonisation of variables between surveys does not take into consideration the methodological differences between the surveys, hence statistical testing between studies was not carried out. PA were measured from reports and may be good to have data supplemented with device-based measures for more in-depth knowledge of bouts of PA during the day, such as in and out of school time. The data were cross-sectional, hence associations between variables can be bi-directional and cannot attest to causal relationships. The opposite associations between children and adolescents in the physical environment factor, may be a reason for future longitudinal studies or trials that examine how the physical environment and PA are related.

## 6 Conclusion

In summary, except gender, sociodemographic inequalities associated with PA were not consistent between children and adolescents. Several findings from the Kazakhstan representative sample contrast findings from other studies from the developed ‘Western’ world. At the individual level, the role of age and BMI categories were surprisingly not associated with children or adolescents. At the family level, increased family wealth was inversely associated with PA. Yet, similar to other studies, intact families and employed parents were positively associated with PA. At the environmental level, positive associations with PA were reported for urban children and rural adolescents. These insights provide nuanced patterns into PA levels of Kazakhstan children and adolescents that contrast with international studies into PA levels and the transition between childhood and adolescence.

## CRediT authorship contribution statement

**Kwok Ng:** Writing – review & editing, Writing – original draft, Methodology, Conceptualization. **Assel Adayeva:** Methodology, Investigation, Formal analysis, Conceptualization. **Shynar Abdrakhmanova:** Writing – review & editing, Writing – original draft, Investigation, Conceptualization. **Stephen Whiting:** Writing – review & editing. **Julianne Williams:** Writing – review & editing. **Tatyana Slazhnyova:** Supervision, Investigation. **Galina Kaussova:** Supervision.

## Declaration of competing interest

The authors declare that they have no known competing financial interests or personal relationships that could have appeared to influence the work reported in this paper.

## Data Availability

The authors do not have permission to share data.

## Footnotes

1 We used the full HBSC dataset, compared to the reduced dataset used in the HBSC international report. We included all students aged 9–16 years from 5th, 7th, 9th grades, as our study examined changes across school grades. The international report had fewer respondents (n = 4731) compared to this study.
